# Genomic analysis of Staphylococcus capitis isolated from blood cultures in neonates at a neonatal intensive care unit in Sweden

**DOI:** 10.1007/s10096-019-03647-3

**Published:** 2019-08-09

**Authors:** Bianca Stenmark, Bengt Hellmark, Bo Söderquist

**Affiliations:** 1 Department of Laboratory Medicine, Faculty of Medicine and Health, Örebro University Hospital, SE-701 85 Örebro, Sweden; 2 School of Health Sciences, Faculty of Medicine and Health, Örebro University, Örebro, Sweden; 3 School of Medical Sciences, Faculty of Medicine and Health, Örebro University, Örebro, Sweden

**Keywords:** Coagulase negative staphylococci, *S. capitis*, NRCS-A clone, Neonatal intensive care unit, Whole-genome sequencing

## Abstract

Emergence of a genetically distinct, multidrug-resistant *Staphylococcus capitis* clone (NRCS-A) present in neonatal intensive care units has recently been extensively reported. The aims of the present study were to investigate which clones of *S. capitis* isolated from blood in a Swedish neonatal intensive care unit (NICU) have been present since 1987 and to investigate whether the NRCS-A clone has disseminated in Sweden. All *S. capitis* isolates from blood cultures of neonates (≤ 28 days of age) between 1987 and 2017 (*n* = 46) were whole-genome sequenced, and core genome multilocus sequence typing (cgMLST) was performed. Single-nucleotide polymorphism (SNP)-based phylogenetic relationships between the *S. capitis* isolates and in silico predictions of presence of genetic traits specific to the NRCS-A clone were identified. Furthermore, antibiotic susceptibility testing, including screening for heterogeneous glycopeptide-intermediate resistance, was performed. Thirty-five isolates clustered closely to the isolates previously determined as belonging to the NRCS-A clone and had fewer than 81 core genome loci differences out of 1063. Twenty-one of these isolates were multidrug resistant. The NRCS-A clone was found in 2001. Six pairs of isolates had differences of fewer than two SNPs. Genetic traits associated with the NRCS-A clone such as *nsr*, *ebh*, *tarJ*, and CRISPR were found in all 35 isolates. The increasing incidence of *S. capitis* blood cultures of neonates is predominantly represented by the NRSC-A clone at our NICU in Sweden. Furthermore, there were indications of transmission between cases; adherence to basic hygiene procedures and surveillance measures are thus warranted.

**Electronic supplementary material:**

The online version of this article (10.1007/s10096-019-03647-3) contains supplementary material, which is available to authorized users.

## Introduction

*Staphylococcus capitis* has been considered a commensal, since it is rarely reported as a pathogen in healthy adults, unless in the presence of cofactors such as foreign bodies or immunosuppression [1] which could be represented by prosthetic valve endocarditis [2] or prosthetic joint infections [3] and bacteraemia in patients with hematological malignancies [4], respectively.

However, *S. capitis* has been shown to cause approximately 20% of all cases of neonatal sepsis at neonatal intensive care units (NICUs) [5, 6]. Recently, the emergence of a genetically distinct, multidrug-resistant (MDR), including methicillin-resistant, *S. capitis* clone has been reported present in NICUs, initially from France but later also from the UK, Belgium, Australia, and New Zealand [7–9]. This specific clone, named NRCS-A, has been thoroughly investigated, and the genomic features of a prototype strain CR01 described [7]. In addition, this *S. capitis* clone also displays reduced susceptibility to last-line antistaphylococcal agents such as vancomycin, both as heteroresistance and resistance [5, 7].

We have previously shown that the incidence of neonatal sepsis due to *S. capitis* has increased from 1987 to 2014 in Örebro County, Sweden, and multilocus sequence typing (MLST) of a limited selection of MDR isolates suggested that these were similar to the isolate CR01 [10]. The dissemination of this clone has been reported from many different countries worldwide [5, 7, 11]. The oldest NRCS-A isolate hitherto described was from Australia in 2000 [7]. However, according to the pulsed-field gel electrophoresis (PFGE) pattern characteristic for the NRCS-A clone, this clone had been found as early as 1994 [11]. Since there are no studies other than ours from Sweden, and limited data from the rest of Scandinavia, the aims of the present study were to investigate which clones of *S. capitis* isolated from blood in a Swedish single-center NICU have dominated since 1987 and to investigate whether the MDR clone NRCS-A has disseminated in Sweden.

## Materials and methods

### Bacterial isolates

All *S. capitis* isolates from blood cultures of neonates (≤ 28 days of age) collected at the Department of Laboratory Medicine, Clinical Microbiology, Örebro University Hospital, Sweden, between 1987 and 2017 (*n* = 46), were included. Before the implementation of the MALDI-TOF MS (Bruker Daltonik, Bremen, Germany) in January 2014, staphylococci were determined by routine methods such as coagulase test, DNase test, and in selected cases, API32Staph (bioMérieux, Marcy l’Étoile, France). All positive blood cultures from 1980 to 2014 were retrospectively determined to species level by MALDI-TOF MS using Microflex LT and Biotyper 3.1 (Bruker Daltonik) [10]. In addition, *S. capitis* isolates from blood cultures from neonates from 2015 to 2017 were included. The isolates were stored in a preservation medium of trypticase soy broth with 0.3% yeast extract and 29% horse serum at − 80 °C.

### Antibiotic susceptibility testing

Antibiotic susceptibility testing was performed on Mueller-Hinton II agar 3.8% *w*/*v* (BD Diagnostic Systems, Sparks, MD, USA) using the standardized disk diffusion method according to the European Committee on Antimicrobial Susceptibility Testing (www.eucast.org) for the following antibiotics: cefoxitin (30 μg), fusidic acid (10 μg), clindamycin (2 μg), erythromycin (15 μg), gentamicin (10 μg), rifampicin (5 μg), trimethoprim-sulfamethoxazole (25 μg), and norfloxacin (10 μg). Isolates resistant to ≥ 3 of the tested antibiotic groups were considered MDR. For vancomycin, minimum inhibitory concentration (MIC) was determined by Etest (bioMérieux).

### Screening for heterogeneous glycopeptide-intermediate *S. capitis*

Detection of heterogeneous glycopeptide-intermediate *S. capitis* (hGISC) was performed using the VAN4 method, the macromethod Etest (MME), and the glycopeptide resistance detection (GRD) Etest as previously described [3, 5, 12, 13]. However, four droplets of 10 μL each, not 10 mL, were used for the VAN4 test.

### Whole-genome sequencing and assembly

DNA extraction was performed using the QIAsymphony DSP Virus/pathogen Midi kit, version 1 (QIAGEN GmbH, Hilden, Germany) on a QIAsymphony (QIAGEN) according to the manufacturer’s description. The protocol was modified to include RNAse treatment as well as Tris-HCl pH 8.0 as elution buffer. The isolates were whole-genome sequenced using the Nextera XT library preparation kit (Illumina Inc., San Diego, CA, USA) on a MiSeq (Illumina) using either v2 2 × 250 bp or v3 2 × 300 paired-end workflow with coverage 40–120×. The reads were trimmed until the average Phred quality was 30 in a window of 20 bases and de novo assembled using Velvet version 1.1.04, using optimized k-mer size within the SeqSphere+ v 4.0.2 software (Ridom GmbH, Münster, Germany). The whole-genome sequence read files have been deposited in the European Nucleotide Archive (ENA) under study accession no. PRJEB32572.

### cgMLST

A core genome multilocus sequence typing (cgMLST) target set, based on genome-wide gene-by-gene comparison, was performed using the cgMLST target definer function (version 4.1.6) in SeqSphere+, using default parameters. *S. capitis* isolate AYP1020 accession number CP007601 (4 April 2018), 2443604 bases, 2262 genes with CDS was used as a reference genome. All publicly available *S. capitis* genomes from NCBI (accessed 8 August 2018), shown in Supplementary Table 1 (*n* = 53), in addition to the genomes from the present study (*n* = 46) were used as query genomes.

### SNP-based phylogenetic analysis

Single-nucleotide polymorphism (SNP)-based phylogenetic relationships between the entire set of *S. capitis* isolates from blood cultures of neonates (*n* = 46) were determined using the REALPHY online tool v1.12 with default parameters. In brief, phylogenetic trees were constructed using single-nucleotide polymorphisms in homologous sites between the assembled contigs of each isolate and the *S. capitis* NRCS-A strain CR01 as reference.

For the SNP-based phylogenetic relationships between the NRCS-A isolates, the reads were trimmed and subsequently mapped to the NRCS-A CR01 genome (ENA accession number LN866849) with CLC Genomics Workbench 11.0 (QIAGEN), using default parameters. The alignments were improved by locally realigning the mapped reads. Indels and structural variants were searched and used as guidance for a second local realignment. The positions used for the SNP tree were determined by the basic variant detection with default parameters (minimum coverage 10 and minimum frequency 35%). Phylogenetic trees were constructed in CLC Genomics Workbench using the SNP tree tool with the maximum likelihood algorithm and the nucleotide substitution model Jukes Cantor with a bootstrap analysis of 100 replicates.

### In silico prediction of presence of NRCS-A-specific traits

In silico predictions of the presence of genes and other traits specific to the NRCS-A clone according to previous studies were performed [8, 14]. All in silico predictions were performed using CLC Genomics Workbench v 11.0. Presence of the *nsr* gene coding for nisin resistance was established using in silico PCR with primers previously described [14]. Presence of the *ebh* gene encoding a cell wall-associated fibronectin-binding protein was determined by mapping the reads of each isolate to AYP1020 Genomic Sequence: NZ_CP007601.1 Range 759600–789017. Presence of *tarJ* encoding teichoic acid biosynthesis was determined by mapping the reads of each isolate to CR01 genomic accession HG737333.1 product accession CDI72760.1. Presence of clustered regularly interspaced short palindromic repeats (CRISPR) was determined using CRISPRCasFinder [15]; evidence of 5 (on a 1–5 scale) was interpreted as present.

## Results

### Antimicrobial susceptibility

**Fig. 1:**
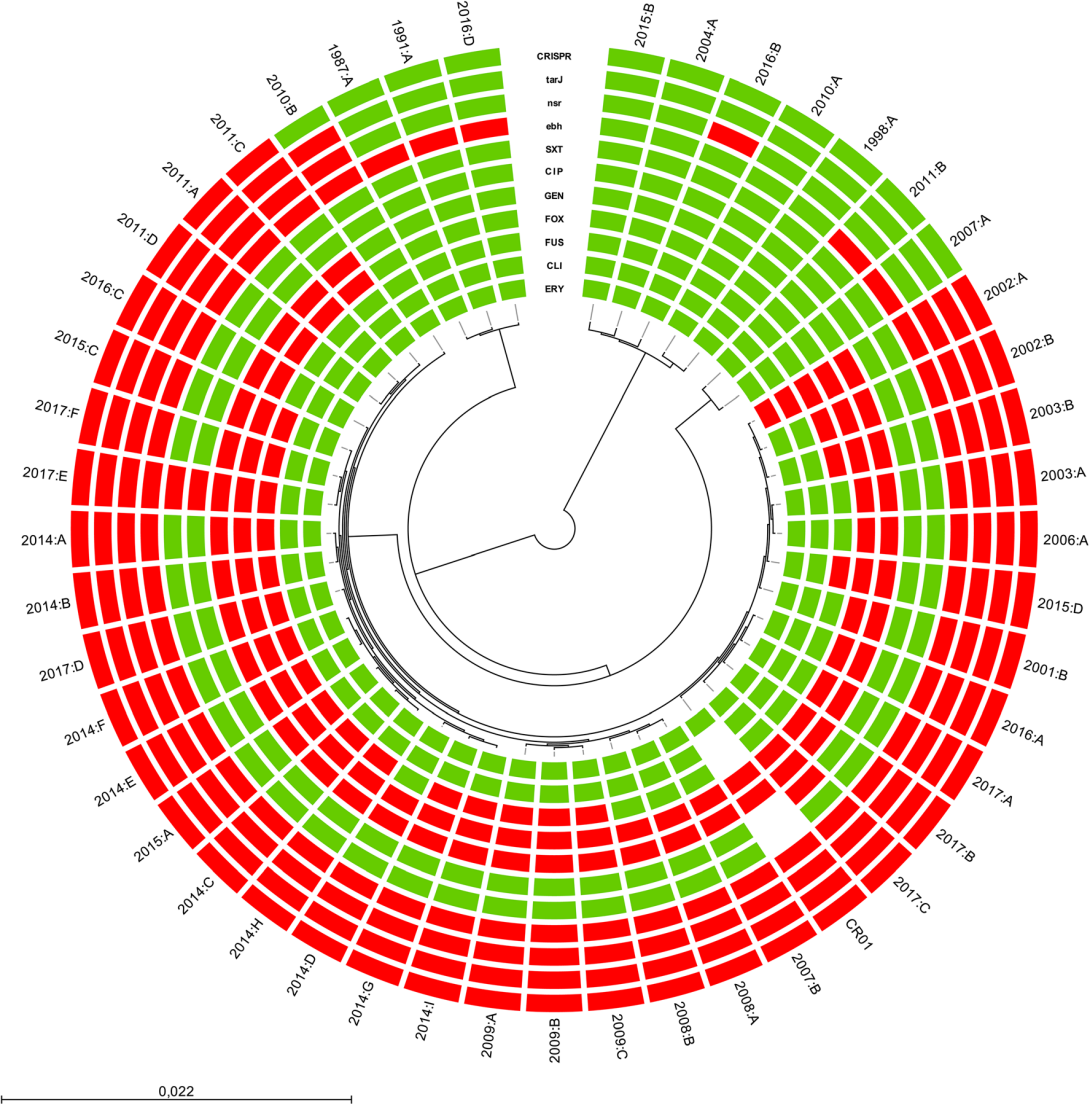
A circular SNP-based maximum likelihood phylogenetic tree using 1,854,749 homologous sites in 46 *S. capitis* isolates from neonates in Örebro County, Sweden (*n* = 46), and NRCS-A isolate CR01 as a reference obtained with the REALPHY v1.12 online tool. Phenotypic resistance to trimethoprim-sulfamethoxazole (SXT), ciprofloxacin (CIP), gentamicin (GEN), cefoxitin (FOX), fusidic acid (FUS), clindamycin (CLI), and erythromycin (ERY) is indicated in red. Genes encoding *tarJ, ebh, nsr*, and CRISPR are shown (red = present, green = absent, and white = no data). The year denotes the year of isolation

Twenty-two isolates out of the 46 isolates (48%) were MDR. The most common resistance profile was a combination of cefoxitin, fusidic acid, and gentamicin resistance (Fig 1). All isolates displaying resistance to cefoxitin indicating methicillin resistance were found to carry the *mecA* gene in the SCC*mec* class V. No isolates displayed decreased susceptibility to vancomycin (median 1.5 mg/L; range 0.25–2 mg/L). However, 29/46 (63%) isolates were identified as hGISC by both MME and GRD Etests, and an additional six isolates displayed hGISC according to the VAN4 method. Among *S. capitis* displaying a MDR phenotype, 17/22 (77%) were hGISC (all three methods). All isolates were susceptible to rifampicin, and only one isolate was resistant to erythromycin and clindamycin. Isolates resistant to erythromycin harbored the *ermC* gene.

### Genome comparison with other international isolates

**Fig. 2:**
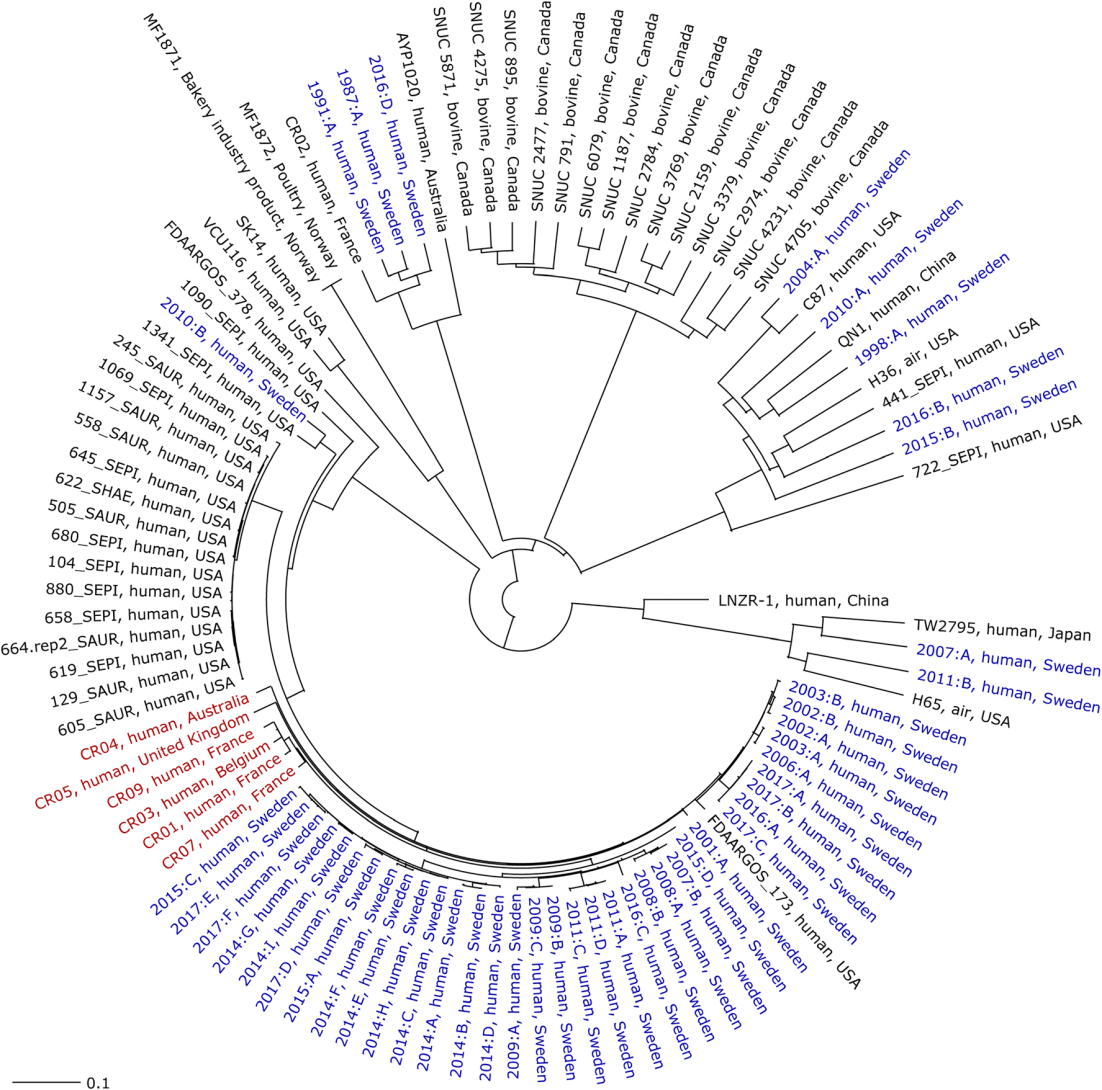
A phylogenetic tree based on the allelic profiles of 1063 cgMLST loci. Publicly available *S. capitis* genomes from NCBI (*n* = 53) and *S. capitis* from Örebro County, Sweden (*n* = 46). Previously described isolates belonging to the NRCS-A clone (*n* = 5) are shown in red [7, 16] and the isolates from neonates in Sweden (*n* = 46) are shown in blue. Isolate name, country of isolation, and host/source are provided for each leaf. The scale bar indicates the percentage of loci with allelic differences out of the 1063 loci

The species-specific cgMLST suitable for global surveillance using 1063 cgMLST loci shows the diversity of the isolates collected from neonates in the present study as well as international *S. capitis* isolates (Fig. 2). Thirty-five isolates clustered closely to the isolates previously determined as belonging to the NICU NRCS-A clone and had fewer than 81 core genome loci differences. The isolates from Sweden were also dispersed among most other clusters; however, these were few and more diverse. Apart from the NRCS-A cluster, only three other isolates from the present study created a cluster of isolates separated by fewer than 100 core genome loci, namely 1991:A, 1987:A, and 2016:D, which clustered with the previously methicillin-susceptible CR02 belonging to pulsotype NRCS-C [16] and isolate AYP1020, which is highly antibiotic susceptible [17]. Isolates from non-human sources clustered separately and only isolates with human hosts clustered within the NRCS-A cluster.

### SNP-based phylogenetic analysis of isolates from Sweden

**Fig. 3:**
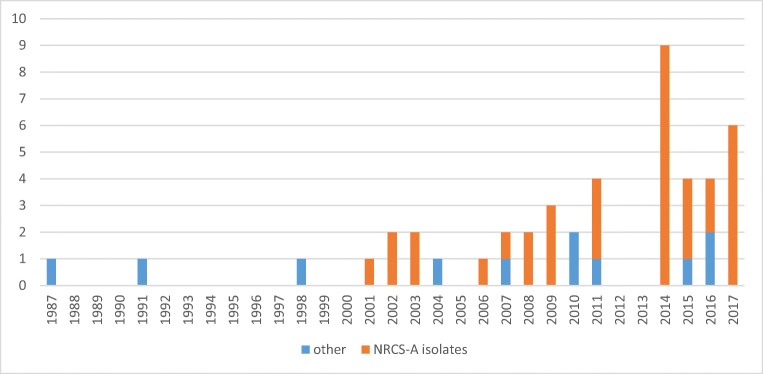
Distribution over time of *S. capitis* isolates belonging to the NRCS-A cluster (*n* = 35) from blood cultures of neonates in Örebro County, Sweden, 1987–2017

SNP analysis was performed to gain more discriminatory power between isolates from the present study. Homologous sites (*n* = 1,854,749) between the 46 isolates aligned to the CR01 reference were used to create the phylogenetic tree in Fig. 1. The SNP analysis showed similar relationships between isolates as in the cgMLST analysis. Multidrug resistance was most common among NRCS-A isolates (21 out of 35 NRCS-A isolates were MDR). No temporal associations were found between clusters, and although the prevalence of the NRCS-A clone seems to have increased in recent years, it was present in Sweden in 2001 (Fig. 3).

Thirty-five out of the 46 isolates were considered to belong to the NRCS-A cluster. Although quite similar, isolate 2010:B was considered a non-NRCS-A isolate because it did not cluster with previously reported NRCS-A isolates (CR01, CR03, CR05, CR07, and CR09) in Fig. 2 and had allelic differences from CR01 in 138 core genome loci as well as 796–848 SNP differences from the other NRCS-A isolates. However, it did harbor the *nsr*, *tarJ*, and *ebh* otherwise unique to NRCS-A isolates (Fig. 1), as previously reported [8, 14]. In general, *nsr*, *ebh*, *tarJ*, and CRISPR were only present in isolates belonging to the NRCS-A cluster (Fig. 1). However, 2016:B did not belong to the NRCS-A cluster but had the *nsr* gene (97.8% sequence identity to CR01), and *ebh* was present in five non-NRCS-A isolates (Fig. 1).

**Fig. 4:**
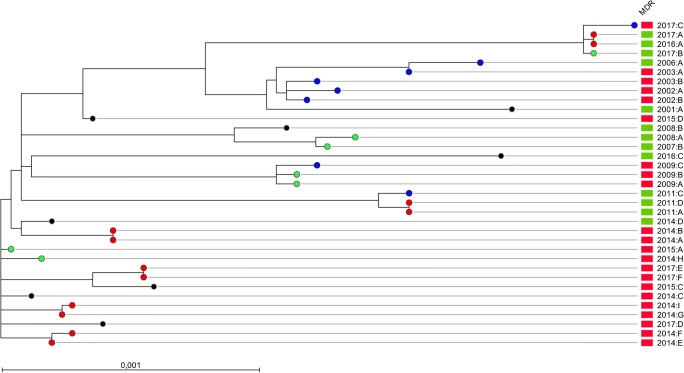
SNP-based maximum likelihood phylogenetic tree using *S. capitis* NRCS-A isolates (*n* = 35) from neonates in Örebro County, Sweden, and CR01 as a reference. Multidrug-resistant isolates are shown with red blocks (*n* = 21). The SNP difference between isolates is indicated with colored dots: 0–2 SNPs in red, 3–5 SNPs in green, 6–10 SNPs in blue, and > 10 SNPs in black

A SNP tree including only the NRCS-A isolates was created to study whether there was any evidence of transmission. There were in total three clusters with identical SNP patterns and another three clusters with 1–2 SNP differences and identical years of collection (Fig. 4), suggesting that these cases were the result of direct transmissions.

## Discussion

The present study showed that the increase of *S. capitis* among neonates with bacteraemia since 2010 in Region Örebro County, Sweden, is mainly due to the dissemination of the MDR *S. capitis* NRCS-A clone; however, this clone was probably introduced as early as in or before 2001. The other isolates that did not belong to the NRCS-A cluster were heterogeneous, and the largest cluster contained only three isolates. Moreover, the SNP phylogenetic analysis provided evidence of direct or indirect transmission of the NRCS-A clone between cases.

Although isolates from distant geographical areas were compared, isolates within the NRCS-A cluster differed in a maximum of 81 out of 1063 cgMLST loci. These results are in concordance with previous studies showing that NRCS-A *S. capitis* is highly conserved and has adapted to the NICU setting [7]. Although the isolates belonging to the NRCS-A clone from Örebro County were highly similar, MDR and non-MDR isolates intermingled within the NRCS-A cluster. The most common MDR profile in the present study was fusidic acid, cefoxitin, and gentamicin resistance, which is in concordance with previous studies [7, 8] where the NICU isolates showed methicillin and aminoglycoside resistance, and either resistance or heteroresistance to vancomycin. None of the *S. capitis* in the present study were resistant to rifampicin, which was in contrast to previous studies reporting on the antimicrobial susceptibility pattern for the NRCS-A clone [5, 7].

A specific characteristic of the NRCS-A clone is harboring the *nsr* gene encoding resistance to nisin, a “broad-spectrum” bacteriocin active against various Gram-positive microorganisms, and the presence of this gene is suggested to affect the establishment of the microbiome of the neonate following birth [14]. All isolates considered to belong to the NRCS-A clone carried the *nsr* gene, and in addition, one isolate not belonging to the clone did carry the *nsr* gene, which displayed a 97.8% sequence identity to the CR01 strain. That isolate was fully susceptible to all tested antibiotics.

In addition, the NRCS-A isolates in the present study harbored *ebh*, *tarJ*, and CRISPR, in concordance with previous studies [8, 14]. However, the *ebh* gene encoding an extracellular matrix-binding protein, and having been described as a potential virulence factor [14], was also present in five non-NRCS-A isolates. This is in concordance with the study by Carter et al. [8], which also found the *ebh* in one isolate from a separate cluster group. Together, these findings could potentially suggest that this gene is not an NRCS-A-specific trait.

The annual numbers of positive blood cultures from neonates yielding coagulase-negative staphylococci (CoNS) have varied considerably during the study period, ranging from 3 to 25 [10], and no cases of *S. capitis* bacteremia were observed in 2012 and 2013. Despite this, there has been an increase in the number of cases with *S. capitis.* The increase in incidence is predominantly represented by the NRSC-A clone that was introduced in the year 2001, indicating an emergence.

Obvious transmission between children seems to have taken place, since there were six pairs of isolates with a difference of fewer than two SNPs between the pair-wise compared isolates. A limitation is the lack of any clinical data regarding the patients from whom these were isolated, as well as any temporal or spatial association. Despite that, it is highly probable that these cases were the result of direct or indirect transmission. This implies the vital importance of adherence to basic hygiene procedures and surveillance measures when the NRSC-A clone has been established at NICUs.

## Electronic supplementary material

ESM 1 (DOCX 19 kb)
