# Supplementary material for: Genomic analysis of Staphylococcus capitis isolated from blood cultures in neonates at a neonatal intensive care unit in Sweden
Source: Eur J Clin Microbiol Infect Dis. 2019 Aug 9;38(11):2069–75. doi: 10.1007/s10096-019-03647-3 (PMC6800862; doi:10.1007/s10096-019-03647-3)
Supplement: Supplementary file 1 — (DOCX 19 kb) [file 10096_2019_3647_MOESM1_ESM.docx]

Supplementary Table 1. All publicly available *S. capitis* genomes from NCBI (accessed 8 August 2018).

| **Strain** | **BioSample** | **BioProject** | **Assembly** | **Size (Mb)** | **WGS** | **Scaffolds** | **Genes** | **Proteins** | **Release Date** | **Modify Date** | **Level** |
| --- | --- | --- | --- | --- | --- | --- | --- | --- | --- | --- | --- |
| AYP1020 | SAMN02739579 | PRJNA232502 | GCA_001028645.1 | 2.50326 | - | 2 | 2414 | 2272 | 2015-06-12 | 2018-04-04 | Complete Genome |
| TW2795 | SAMD00041516 | PRJDB4225 | GCA_002356175.1 | 2.48678 | - | 2 | 2447 | 2325 | 2017-02-01 | 2017-09-27 | Complete Genome |
| FDAARGOS_378 | SAMN07312422 | PRJNA231221 | GCA_002591175.1 | 2.48515 | - | 2 | 2414 | 2272 | 2017-10-18 | 2017-10-20 | Complete Genome |
| C87 | SAMN02463752 | PRJNA38749 | GCA_000183705.1 | 2.47361 | ACRH01 | 14 | 2452 | 2239 | 2010-11-30 | 2017-03-30 | Scaffold |
| CR01 | SAMEA3138827 | PRJEB1494 | GCA_000499705.1 | 2.50377 | CBUB01 | 8 | 2419 | 2298 | 2013-10-30 | 2017-04-02 | Scaffold |
| SK14 | SAMN00000713 | PRJNA31013 | GCA_000174135.1 | 2.43583 | ACFR01 | 32 | 2401 | 2272 | 2009-02-03 | 2017-11-15 | Contig |
| VCU116 | SAMN00116853 | PRJNA53751 | GCA_000221525.2 | 2.45257 | AFTX01 | 38 | 2413 | 2296 | 2011-07-25 | 2017-11-22 | Contig |
| QN1 | SAMN02471139 | PRJNA159795 | GCA_000263775.1 | 2.4301 | AJTH01 | 30 | 2403 | 2231 | 2012-05-22 | 2017-11-23 | Contig |
| LNZR-1 | SAMN02680303 | PRJNA240935 | GCA_000712995.1 | 2.59586 | JGYJ01 | 90 | 2487 | 2428 | 2014-06-27 | 2017-04-02 | Contig |
| 1157_SAUR | SAMN03197113 | PRJNA267549 | GCA_001062085.1 | 2.55353 | JWAZ01 | 56 | 2440 | 2362 | 2015-07-10 | 2017-04-03 | Contig |
| 558_SAUR | SAMN03197757 | PRJNA267549 | GCA_001063695.1 | 2.58486 | JVCF01 | 40 | 2478 | 2392 | 2015-07-10 | 2017-04-03 | Contig |
| 1090_SEPI | SAMN03197053 | PRJNA267549 | GCA_001069195.1 | 2.4232 | JWDH01 | 70 | 2282 | 2228 | 2015-07-10 | 2017-04-03 | Contig |
| 1069_SEPI | SAMN03197031 | PRJNA267549 | GCA_001070105.1 | 2.54173 | JWED01 | 76 | 2423 | 2351 | 2015-07-10 | 2017-04-03 | Contig |
| 680_SEPI | SAMN03197883 | PRJNA267549 | GCA_001073915.1 | 2.52573 | JUXJ01 | 167 | 2420 | 2330 | 2015-07-10 | 2017-04-03 | Contig |
| 880_SEPI | SAMN03198086 | PRJNA267549 | GCA_001074775.1 | 2.52276 | JUPO01 | 114 | 2412 | 2339 | 2015-07-10 | 2017-04-03 | Contig |
| 722_SEPI | SAMN03197927 | PRJNA267549 | GCA_001074935.1 | 2.34419 | JUVR01 | 158 | 2284 | 2123 | 2015-07-10 | 2017-04-03 | Contig |
| CR03 | SAMEA3358846 | PRJEB9122 | GCA_001179785.1 | 2.50535 | CVUF01 | 31 | 2424 | 2305 | 2015-06-19 | 2017-04-03 | Contig |
| CR04 | SAMEA3271079 | PRJEB8618 | GCA_001201015.1 | 2.50859 | CTEM01 | 38 | 2407 | 2309 | 2015-03-25 | 2017-04-03 | Contig |
| CR05 | SAMEA3271080 | PRJEB8618 | GCA_001220005.1 | 2.54012 | CTEO01 | 39 | 2459 | 2356 | 2015-03-26 | 2017-04-03 | Contig |
| CR09 | SAMEA3271081 | PRJEB8618 | GCA_001220605.1 | 2.48716 | CTEL01 | 34 | 2403 | 2286 | 2015-03-25 | 2017-04-03 | Contig |
| FDAARGOS_173 | SAMN03996314 | PRJNA231221 | GCA_001471555.2 | 2.60255 | LORZ02 | 4 | 2560 | 2408 | 2015-12-21 | 2018-01-23 | Contig |
| H65 | SAMN04621680 | PRJNA317599 | GCA_001650395.1 | 2.49343 | LWCP01 | 45 | 2434 | 2295 | 2016-05-24 | 2017-04-05 | Contig |
| H36 | SAMN04621681 | PRJNA317600 | GCA_001650475.1 | 2.41844 | LWCQ01 | 31 | 2391 | 2197 | 2016-05-24 | 2017-04-05 | Contig |
| MF1871 | SAMN04479465 | PRJNA311173 | GCA_001651285.1 | 2.46331 | LSKZ01 | 46 | 2394 | 2295 | 2016-05-25 | 2017-04-05 | Contig |
| MF1872 | SAMN04479466 | PRJNA311173 | GCA_001651335.1 | 2.47154 | LSLA01 | 45 | 2397 | 2297 | 2016-05-25 | 2017-04-05 | Contig |
| SNUC 6079 | SAMN06172863 | PRJNA342349 | GCA_003039855.1 | 2.43409 | PZCT01 | 113 | 2381 | 2230 | 2018-04-05 | 2018-04-11 | Contig |
| SNUC 5871 | SAMN06172861 | PRJNA342349 | GCA_003039875.1 | 2.43426 | PZCU01 | 73 | 2370 | 2239 | 2018-04-05 | 2018-04-11 | Contig |
| SNUC 4231 | SAMN06172857 | PRJNA342349 | GCA_003039895.1 | 2.45705 | PZCX01 | 162 | 2407 | 2255 | 2018-04-05 | 2018-04-11 | Contig |
| SNUC 4705 | SAMN06172859 | PRJNA342349 | GCA_003040535.1 | 2.41763 | PZCV01 | 356 | 2379 | 2178 | 2018-04-05 | 2018-04-11 | Contig |
| SNUC 4275 | SAMN06172858 | PRJNA342349 | GCA_003040555.1 | 2.44527 | PZCW01 | 158 | 2388 | 2236 | 2018-04-05 | 2018-04-11 | Contig |
| SNUC 3769 | SAMN06172855 | PRJNA342349 | GCA_003040575.1 | 2.41772 | PZCY01 | 68 | 2355 | 2215 | 2018-04-05 | 2018-04-11 | Contig |
| SNUC 3379 | SAMN06172854 | PRJNA342349 | GCA_003040595.1 | 2.4217 | PZCZ01 | 49 | 2354 | 2232 | 2018-04-05 | 2018-04-11 | Contig |
| SNUC 2784 | SAMN06172851 | PRJNA342349 | GCA_003040615.1 | 2.41895 | PZDB01 | 132 | 2362 | 2216 | 2018-04-05 | 2018-04-11 | Contig |
| SNUC 2159 | SAMN06172848 | PRJNA342349 | GCA_003040635.1 | 2.43098 | PZDD01 | 52 | 2372 | 2236 | 2018-04-05 | 2018-04-11 | Contig |
| SNUC 1187 | SAMN06172845 | PRJNA342349 | GCA_003040655.1 | 2.40854 | PZDE01 | 79 | 2333 | 2208 | 2018-04-05 | 2018-04-11 | Contig |
| SNUC 895 | SAMN06172844 | PRJNA342349 | GCA_003040675.1 | 2.41635 | PZDF01 | 207 | 2367 | 2199 | 2018-04-05 | 2018-04-25 | Contig |
| SNUC 2477 | SAMN06172849 | PRJNA342349 | GCA_003041035.1 | 2.42095 | PZDC01 | 160 | 2383 | 2226 | 2018-04-05 | 2018-04-11 | Contig |
| SNUC 2974 | SAMN06172852 | PRJNA342349 | GCA_003041055.1 | 2.52797 | PZDA01 | 100 | 2475 | 2330 | 2018-04-05 | 2018-04-11 | Contig |
| SNUC 791 | SAMN06172842 | PRJNA342349 | GCA_003041075.1 | 2.41558 | PZDG01 | 40 | 2352 | 2221 | 2018-04-05 | 2018-04-11 | Contig |
| CR07 | SAMEA3661573 | PRJEB8890 | GCA_900068995.1 | 2.4746 | CZWH01 | 26 | 2406 | 2301 | 2015-11-14 | 2017-06-09 | Contig |
| 129_SAUR | SAMN03197261 | PRJNA267549 | GCA_001060815.1 | 2.59971 | JVVH01 | 194 | 2445 | 2377 | 2015-07-10 | 2017-04-03 | Scaffold |
| 245_SAUR | SAMN03197435 | PRJNA267549 | GCA_001064095.1 | 2.53678 | JVOP01 | 120 | 2441 | 2343 | 2015-07-10 | 2017-04-03 | Scaffold |
| 505_SAUR | SAMN03197698 | PRJNA267549 | GCA_001065245.1 | 2.57911 | JVEM01 | 52 | 2474 | 2393 | 2015-07-10 | 2017-04-03 | Scaffold |
| 605_SAUR | SAMN03197805 | PRJNA267549 | GCA_001065645.1 | 2.53814 | JVAJ01 | 82 | 2417 | 2352 | 2015-07-10 | 2017-04-03 | Scaffold |
| 664.rep2_SAUR | SAMN03197868 | PRJNA267549 | GCA_001066795.1 | 2.56104 | JUXY01 | 40 | 2451 | 2369 | 2015-07-10 | 2017-04-03 | Scaffold |
| 104_SEPI | SAMN03197005 | PRJNA267549 | GCA_001069155.1 | 2.55798 | JWFD01 | 43 | 2446 | 2367 | 2015-07-10 | 2017-04-03 | Scaffold |
| 1341_SEPI | SAMN03197323 | PRJNA267549 | GCA_001069765.1 | 2.56112 | JVSX01 | 75 | 2445 | 2376 | 2015-07-10 | 2017-04-03 | Scaffold |
| 441_SEPI | SAMN03197633 | PRJNA267549 | GCA_001071095.1 | 2.3924 | JVGZ01 | 56 | 2324 | 2176 | 2015-07-10 | 2017-04-03 | Scaffold |
| 619_SEPI | SAMN03197819 | PRJNA267549 | GCA_001073565.1 | 2.54248 | JUZV01 | 57 | 2428 | 2350 | 2015-07-10 | 2017-04-03 | Scaffold |
| 622_SHAE | SAMN03197822 | PRJNA267549 | GCA_001073605.1 | 2.54034 | JUZS01 | 63 | 2433 | 2351 | 2015-07-10 | 2017-04-03 | Scaffold |
| 645_SEPI | SAMN03197846 | PRJNA267549 | GCA_001073715.1 | 2.55465 | JUYU01 | 40 | 2467 | 2360 | 2015-07-10 | 2017-04-03 | Scaffold |
| 658_SEPI | SAMN03197860 | PRJNA267549 | GCA_001073835.1 | 2.54184 | JUYG01 | 64 | 2429 | 2348 | 2015-07-10 | 2017-04-03 | Scaffold |
| CR03 | SAMEA3271078 | PRJEB8618 | GCA_001215085.1 | 2.50835 | CTEB01 | 1 | 2427 | 2299 | 2015-03-25 | 2017-04-03 | Scaffold |
| CR02 | SAMEA3661572 | PRJEB8891 | GCA_001458675.1 | 2.34475 | CZWI01 | 320 | 2415 | 2295 | 2015-11-14 | 2015-11-14 | Contig |
